# Retraction Note: AMLnet, A deep-learning pipeline for the differential diagnosis of acute myeloid leukemia from bone marrow smears

**DOI:** 10.1186/s13045-024-01582-1

**Published:** 2024-08-01

**Authors:** Zebin Yu, Jianhu Li, Xiang Wen, Yingli Han, Penglei Jiang, Meng Zhu, Minmin Wang, Xiangli Gao, Dan Shen, Ting Zhang, Shuqi Zhao, Yijing Zhu, Jixiang Tong, Shuchong Yuan, HongHu Zhu, He Huang, Pengxu Qian

**Affiliations:** 1 Center for Stem Cell and Regenerative Medicine and Bone Marrow Transplantation Center of the First Affiliated Hospital, Zhejiang University School of Medicine, Hangzhou, 310058 China; 2 Liangzhu Laboratory, Zhejiang University Medical Center, 1369 West Wenyi Road, Hangzhou, China; 3 Institute of Hematology, Zhejiang Engineering Laboratory for Stem Cell and Immunotherapy, Zhejiang University, Hangzhou, 310058 China; 4 Department of Hematology, The First Affiliated Hospital, Zhejiang University School of Medicine, Hangzhou, Zhejiang China; 5 College of Computer Science and Technology at Zhejiang University, Hangzhou, Zhejiang China; 6 Department of Hematology, Hangzhou First People’s Hospital, Zhejiang University School of Medicine, Hangzhou, Zhejiang China; 7 Bone Marrow Transplantation Center, The First Affiliated Hospital, Zhejiang University School of Medicine, Hangzhou, China

**Journal of Hematology & Oncology (2023) 16:27**

10.1186/s13045-023-01419-3.

The Editor-in-Chief has retracted this article because the authors were unable to provide documentary evidence that they received ethics approval for the work reported.

Xiangli Gao, Dan Shen, Ting Zhang, Shuqi Zhao, Yijing Zhu, Jixiang Tong, and Shuchong Yuan agree with this retraction. The remaining authors did not respond to correspondence from the Publisher about this retraction.

